# Discrepant Fibrinolytic Response in Plasma and Whole Blood during Experimental Endotoxemia in Healthy Volunteers

**DOI:** 10.1371/journal.pone.0059368

**Published:** 2013-03-15

**Authors:** Sisse R. Ostrowski, Ronan M. G. Berg, Nis A. Windeløv, Martin A. S. Meyer, Ronni R. Plovsing, Kirsten Møller, Pär I. Johansson

**Affiliations:** 1 Section for Transfusion Medicine, Capital Region Blood Bank, Rigshospitalet, Copenhagen, Denmark; 2 Centre of Inflammation and Metabolism, Dept. of Infectious Diseases, Section M7641, Rigshospitalet, Copenhagen, Denmark; 3 Department of Anesthesia, Centre of Head and Orthopedics, Rigshospitalet, Copenhagen, Denmark; 4 Intensive Care Unit, Section 4131, Department of Anesthesia, Rigshospitalet, Copenhagen, Denmark; 5 Neurointensive Care Unit, Department of Neuroanesthesiology, Rigshospitalet, Copenhagen, Denmark; 6 Department of Surgery, Center for Translational Injury Research (CeTIR), University of Texas Medical School at Houston, Houston, Texas, United States of America; Beth Israel Deaconess Medical Center, United States of America

## Abstract

**Background:**

Sepsis induces early activation of coagulation and fibrinolysis followed by late fibrinolytic shutdown and progressive endothelial damage. The aim of the present study was to investigate and compare the functional hemostatic response in whole blood and plasma during experimental human endotoxemia by the platelet function analyzer, Multiplate and by standard and modified thrombelastography (TEG).

**Methods:**

Prospective physiologic study of nine healthy male volunteers undergoing endotoxemia by means of a 4-hour infusion of E. coli lipopolysaccharide (LPS, 0.5 ng/kg/hour), with blood sampled at baseline and at 4 h and 6 h. Physiological and standard biochemical data and coagulation tests, TEG (whole blood: TEG, heparinase-TEG, Functional Fibrinogen; plasma: TEG±tissue-type plasminogen activator (tPA)) and Multiplate (TRAPtest, ADPtest, ASPItest, COLtest) were recorded. Mixed models with Tukey post hoc tests and correlations were applied.

**Results:**

Endotoxemia induced acute SIRS with increased HR, temperature, WBC, CRP and procalcitonin and decreased blood pressure. It also induced a hemostatic response with platelet consumption and reduced APTT while INR increased (all p<0.05). Platelet aggregation decreased (all tests, p<0.05), whereas TEG whole blood clot firmness increased (G, p = 0.05). Furthermore, during endotoxemia (4 h), whole blood fibrinolysis increased (clot lysis time (CLT), p<0.001) and Functional Fibrinogen clot strength decreased (p = 0.049). After endotoxemia (6 h), whole blood fibrinolysis was reduced (CLT, p<0.05). In contrast to findings in whole blood, the plasma fibrin clot became progressively more resistant towards tPA-induced fibrinolysis at both 4 h and 6 h (p<0.001).

**Conclusions:**

Endotoxemia induced a hemostatic response with reduced primary but enhanced secondary hemostasis, enhanced early fibrinolysis and fibrinogen consumption followed by downregulation of fibrinolysis, with a discrepant fibrinolytic response in plasma and whole blood. The finding that blood cells are critically involved in the vasculo-fibrinolytic response to acute inflammation is important given that disturbances in the vascular system contribute significantly to morbidity and mortality in critically ill patients.

## Introduction

Experimental human endotoxemia induced by intravenous administration of purified standard reference lipopolysaccharide (LPS) to healthy volunteers induces an acute systemic inflammatory response, which mimics the inflammatory response of early sepsis as well as other acute inflammatory conditions [1]–[3]. Sepsis is associated with initial activation of coagulation and fibrinolysis followed by late fibrinolytic shutdown and exhaustion of the natural anticoagulant systems [4], the latter mainly due to progressive endothelial disruption and damage [5], [6]. Conventional coagulation tests like activated partial thromboplastin time (APTT), international normalized ratio (INR), platelet count, plasma fibrinogen and D-dimer neither reveal (changes in) fibrinolysis nor platelet (dys)function, both of which may contribute significantly to hyper- and hypocoagulability in critically ill patients. However, viscoelastic hemostatic whole blood tests, such as Thrombelastography (TEG®, Hemoscope–Hemonetics, Niles, IL, US) and Rotation Thromboelastometry (ROTEM®, TEM Inc., Durham, NC, US) [7] as well as platelet function tests, such as light transmission aggregometry and whole blood platelet function tests (e.g. Multiple Platelet function Analyzer; Multiplate®, Verum Diagnostica GmbH, Munich, Germany) [8] reveal both fibrinolysis and platelet function. Several studies have characterized the coagulopathy in sepsis [9]–[22] and experimental human [23], [24] and animal [25]–[29] endotoxemia by these tests.

In addition to fibrinolytic changes and platelet dysfunction, degradation of the endothelial glycocalyx may, through release of large amounts of heparin-like substances [30], [31], induce endogenous heparinization that contributes to hypocoagulability in septic patients 32–37, and we recently reported similar findings in severely injured trauma patients [38].

Excessive sympathoadrenal activation is a hallmark of acute critical illness and the accompanying increase in circulating catecholamines induces widespread dose-dependent effects on metabolism and the vascular system [39]–[41]. Apparently, catecholamines induce opposite directed effects on the endothelium (progressive activation and damage) [42]–[44] and circulating blood (initial hypercoagulability followed by progressive hypocoagulability, hyperfibrinolysis and endogenous heparinization) [38], [42], [45]–[49] and we infer that this reflects an evolutionary adapted response aiming at maintaining blood flow through a damaged and procoagulant microvasculature in the (shocked) critically ill patient [42]. Similarly to the opposite directed effects of catecholamines on the vascular endothelium and circulating blood [38], [42]–[49], the cells and fluid that constitutes the fluid phase of the vascular system i.e., the circulating blood, may also respond in opposite directions in acute inflammatory conditions in order to maintain homeostasis in the vascular system. In vitro studies of platelet function in sepsis and experimental endotoxemia have revealed that addition of septic plasma to control platelets can induce a pathologic response [28], [50], and that removal of septic plasma from patient platelets can restore platelet function [28]. Also, fibrinolytic activity may both be enhanced and inhibited by circulating blood cells and cell-derived microparticles [51]–[55]. Thus, platelets protect the clot against tissue-type plasminogen activator (tPA)-induced fibrinolysis [51] and enhance the antifibrinolytic effect of exogenous FXIII in vitro [52] and red blood cells confer lytic resistance to fibrin resulting from modified fibrin structure and impaired plasminogen activation [56]. In contrast, other blood and endothelial cells, and their derived microparticles, promote fibrinolysis through the action of cell-associated urokinase-type plasminogen activator (uPA) and tPA [53]–[55] and leukocytes promote fibrinolysis through various alternative pathways [57]. Together, this emphasizes that platelets and other blood cells are critically involved in balancing the vasculo-fibrinolytic response, which may contribute to discrepant findings when evaluating fibrinolysis in plasma and whole blood. Given that sepsis and other acute inflammatory conditions are characterized by excessive changes in hemostasis and the vascular system that may be differently driven by and detected in whole blood and plasma, improved understanding and characterization of this response is of critical importance for optimizing and potentially goal-directing therapy.

The primary aim of the present study was to investigate and compare the functional hemostatic response in whole blood and plasma during experimental human endotoxemia as evaluated by Multiplate and by standard and modified TEG analyses, respectively. We expected that endotoxemia would induce a hemostatic response comparable to that previously described [23], [24] and given the role of blood cells for fibrinolysis [51]–[55], we hypothesized that fibrinolytic activity during endotoxemia would differ in plasma and whole blood.

Here we report that experimental endotoxemia by means of a 4-hour 0.5 ng/kg/hour LPS-infusion in healthy volunteers induced a hemostatic response with reduced primary but enhanced secondary hemostasis, enhanced early fibrinolysis and fibrinogen consumption followed by downregulation of fibrinolysis, with a discrepant fibrinolytic response in plasma and whole blood. Our findings indicate a critical role of platelets and/or other blood cells in the vasculo-fibrinolytic response to acute inflammation. The finding here that platelets and other blood cells, not present in plasma, may enhance fibrinolysis in acute inflammatory conditions is important given that especially thrombocytopenia is a strong predictor of poor outcome including excessive non-bleeding mortality in critically ill patients [58]–63. Since thrombocytopenia in septic patients often coexists with bleeding and excessive thrombus formation, or even overt disseminated intravascular coagulation (DIC) [59], it is tempting to speculate that thrombocytopenia may directly contribute to tip the hemostatic balance in the circulating blood towards a reduction in fibrinolysis and thereby paradoxically contribute to enhanced thrombus formation in the microvasculature.

## Materials and Methods

### Experimental human endotoxemia

The study was approved by the Scientific Ethical Committee of Copenhagen and Frederiksberg Municipalities, Denmark (file number H-A-2009-020 with amendments) and The Danish Data Protection Agency and was performed in accordance with the Declaration of Helsinki.

Nine healthy male volunteers (mean±SD age 23±2 years) were enrolled after giving oral and written informed consent. All had an unremarkable medical history without signs of infection within 4 weeks ahead of the trial day and none took any regular medication. Before inclusion, volunteers underwent a thorough physical examination, a 12-lead electrocardiogram (ECG) was obtained and standard laboratory tests were performed. All tests were normal.

Endotoxemia: Following 12-hour overnight fast volunteers reported to the intensive care unit (ICU) at 7∶30 am and were placed in bed. They were catheterized with an intravenous antecubital catheter (for LPS-infusion) and following local anesthesia (lidocaine, 20 mg/ml) an arterial line was inserted in the left radial artery. Heart rate (via a three-lead ECG), invasive blood pressure (mean arterial pressure, MAP) and peripheral oxygen saturation (SpO_2_, by pulse oximetry) were continuously monitored and medically qualified personnel were present at all times. Experimental endotoxemia was induced by means of a 4-hour continuous intravenous infusion of purified *Escherichia coli* LPS (infusion rate 0.5 ng/kg/hour; Batch G2 B274, US Pharmacopeial Convention, Rockville, Maryland, US). In this model, plasma tumor necrosis factor (TNF)-α reaches its peak value at approximately 1 hour after cessation of the infusion [3], [64]. As part of another sub-study, which investigated cerebral auto regulation (data reported elsewhere), noradrenaline was infused immediately after blood samples had been collected at baseline and 4 h as previously described [65]. Given the short in vivo half-life of noradrenaline (t½ = 2.5 min in healthy individuals [66]) this drug could be assumed to be completely metabolized at the following blood sample time-points.

Blood was sampled for routine biochemistry and functional hemostatic measurements (TEG, Multiplate) at 0 h, 4 h and 6 h where 0 h denotes the time when LPS-infusion was initiated; thus 4 h corresponds to the cessation of the infusion (denoted “during endotoxemia”) and 6 h is two hours after cessation. Volunteers were allowed to drink tap water ad libitum during the study day. They were discharged after 12 hours following removal of catheters and a light meal. According to the Danish Legislation, volunteers were not allowed payment alone for their attendance in the study. However, they were compensated for the loss of earnings and for any pain and suffering associated with the study.

### Blood samples and routine biochemistry

Blood was sampled from the radial artery catheter, to mimic the conditions under which blood is often sampled in the critically ill patient. Routine biochemistry were analyzed in a DS/EN ISO 15189 standardized laboratory as follows: Blood cell counts (XE-2100, Sysmex, Japan), C-reactive protein (CRP) (Modular P-modul, Roche, Switzerland), procalcitonin (IMFA, Kryptor, Immulite), D-dimer, fibrinogen, enzymatic active antithrombin (AT), activated partial thromboplastin time (APTT), international normalized ratio (INR) and coagulation factor II-VII-X (ACL TOP, Beckman Coulter, Inc., CA, US), ABG and lactate (Radiometer ABL 725/735, Copenhagen, Denmark).

### Multiplate

Platelet aggregation in heparinized whole blood was analyzed by impedance aggregometry using a Multiple Platelet function Analyzer (Multiplate® analyzer, Dynabyte GmbH), applying commercially available platelet agonists, according to the manufacturers recommendations [normal range reported by Dynabyte GmbH]: TRAPtest (thrombin-receptor activating peptide (TRAP)-6 32 µM [92–151 U]), ADPtest (ADP 6.5 µM [55–117 U]), ASPItest (arachidonic acid 0.5 mM [79–141 U]) and COLtest (collagen 3.2 µg/ml [61–108 U]). Results of each test were recorded as aggregation units (U) or as U per platelet (U/10^9^ platelets, U divided by platelet count).

### Thrombelastography (TEG)

TEG whole blood clot formation was evaluated simultaneously in 3.2% citrated blood samples by kaolin-activated (TEG), kaolin-heparinase-activated (heparinase-TEG) and tissue-factor (TF)-activated platelet-blocked (TEG Functional Fibrinogen®, FF) analyses by a TEG® 5000 Hemostasis Analyzer System (Haemonetics Corp., MA, US), according to the manufacturers recommendations. All analyses were conducted at 37°C. The simultaneous TEG and heparinase-TEG analysis allowed investigation of endogenous heparinization as previously described [38] and the FF analysis allowed investigation of fibrinogen contribution to TEG clot strength. The variables recorded were [normal TEG range reported by Haemonetics Corp.]: Reaction time (R [3–8 min], rate of initial fibrin formation), angle (α [55–78 degrees], clot growth kinetics), clot strength (maximum amplitude (MA) [51–69 mm], maximum clot strength; shear elastic modulus strength G [4,600–10,900 dyn/cm^2^], global clot strength; FF MA [14–24 mm], fibrinogen clot strength) and fibrinolysis (clot lysis time (CLT [min], velocity of clot degradation reflecting fibrinolysis; Ly30/60 (%), percent lysis 30/60 min after MA)[7]. To estimate platelet contribution to clot strength (MA) we calculated platelet MA (mm) by subtracting FF MA from TEG MA, with results reported as crude platelet MA and platelet MA per platelet (mm/10^9^ platelets, platelet MA divided by platelet count).

The day-to-day coefficient of variation of whole blood TEG MA is <7% in our laboratory [67].

In addition, the clotting potential of citrated plasma i.e., the capacity for formation of a pure fibrin clot was investigated by TEG®. Briefly, citrated plasma samples were thawed from −80°C just before analysis and 340 µl plasma was recalcified (20 µl 0.2 M CaCl_2_, final concentration 11.1 mM) and activated with TF (lipidated recombinant human TF, Innovin, Dade Behring, Marburg, Germany; final dilution 1∶42,500) and analyzed immediately at 37°C. To assess the clot resistance to fibrinolysis, citrated plasma samples were analyzed with or without addition of 1.8 nM tissue-type plasminogen activator (tPA, single-chain, American Diagnostica, Greenwich, US) as previously described [67]. The TEG variables described above were recorded.

### Statistics

Statistical analysis was performed using SAS 9.1 (SAS Institute Inc., Cary, NC, US). Data from volunteers were investigated by repeated-measures (RM) analyses (PROC MIXED, autoregressive covariance structure, SAS) and Tukey post hoc tests. Goodness of fit of the mixed model was assessed by investigating the residuals. Correlations between variables were investigated by Pearson correlations, and reported by R and p-values. Data are presented as means ±SD. P-values <0.05 were considered significant.

## Results

### Physiology, inflammation and routine coagulation tests

Endotoxemia induced a systemic inflammatory response syndrome (SIRS) as indicated by increased HR, temperature and white blood cell count and decreased MAP (Table 1). Furthermore, lymphocyte and monocyte counts decreased while CRP and procalcitonin increased. With regard to routine coagulation tests, platelet count, APTT and factor II, VII and X decreased and INR increased whereas plasma fibrinogen, D-dimer and antithrombin did not change (Table 1).

**Table 1:** Physiology and standard biochemistry in nine healthy volunteers before (0 h), during (4 h) and after (6 h) induction of experimental endotoxemia by means of a 4 h 0.5 ng/kg/hour LPS-infusion.

		Endotoxemia (n = 9)				
	Units	0 h	4 h	6 h	RM p-value	Tukey
**Physiology**						
HR	bpm	58±7	94±7	97±15	**<0.001**	^a,b^
MAP	mmHg	92±7	78±9	86±14	**0.020**	^a^
SpO_2_	%	0.98±0.00	0.97±0.01	0.97±0.01	0.090	
Temperature	°C	36.6±0.3	39.0±0.3	38.6±0.3	**<0.001**	^a,b^
SBE	mmol/l	1.1±1.1	0.3±1.2	0.8±1.3	**0.003**	^a^
pH		7.41±0.02	7.45±0.03	7.45±0.01	**<0.001**	^a,b^
Lactate	mmol/l	0.9±0.4	1.0±0.4	1.0±0.2	**0.049**	^b^
**Inflammation**						
WBC	10^9^/l	5.4±0.9	6.8±3.0	12.4±2.7	**<0.001**	^b,c^
Neutrophils	10^9^/l	3.1±0.9	6.1±2.8	11.7±2.5	**<0.001**	^a,b,c^
Progenitor cells	10^9^/l	0.014±0.005	0.017±0.011	0.040±0.017	**<0.001**	^b,c^
Lymphocytes	10^9^/l	1.7±0.5	0.6±0.3	0.4±0.1	**<0.001**	^a,b^
Monocytes	10^9^/l	0.42±0.11	0.05±0.03	0.29±0.17	**<0.001**	^a,c^
CRP	mg/l	1.1±0.3	1.3±0.6	2.2±1.1	**0.012**	^b^
Procalcitonin	µg/l	0.1±0.0	ND	6.2±3.1	**<0.001**	^b^
Hemoglobin	mmol/l	8.9±0.7	8.8±0.7	8.9±0.7	NS	
**Routine coagulation**						
Platelets	10^9^/l	218±35	188±32	177±31	<0.001	^a,b^
Fibrinogen	g/l	2.0±0.2	2.0±0.2	2.0±0.2	NS	
D-dimer	mg/l	0.2±0.1	2.8±4.7	1.5±1.2	0.162	
AT	10^3^ IU	1.02±0.07	0.99±0.06	1.01±0.07	0.102	
APTT	Sec	30±4	23±2	23±2	**<0.001**	^a,b^
INR	Ratio	1.1±0.1	1.2±0.1	1.2±0.1	**<0.001**	^a,b^
Factor II-VII-X	U	0.8±0.1	0.7±0.1	0.7±0.1	**<0.001**	^a,b^

Data are presented as means±SD. Data from volunteers were compared by repeated-measures analyses (RM) and Tukey post hoc tests: p<0.05 for 0 h vs. 4 h^a^, 0 h vs. 6 h^b^ and 4 h vs. 6 h^c^. P-values <0.2 are shown and in bold if p<0.05.

HR, heart rate; MAP, mean arterial blood pressure; SpO_2_, peripheral oxygen saturation; SBE, standard base excess; WBC, white blood cells; CRP, c-reactive protein; AT, antithrombin; APTT, activated partial thromboplastin time; INR, international normalized ratio. NS, non-significant; ND, not done.

### Hemostatic response in whole blood

Endotoxemia induced a decline in primary hemostasis (platelet adhesion and aggregation) as evaluated by Multiplate® TRAPtest, ADPtest, ASPItest and COLtest and it significantly reduced the per platelet response in the TRAPtest (Table 2).

**Table 2:** Functional hemostatic assays in whole-blood (impedance aggregometry (Multiplate), Thrombelastography (TEG), Functional fibrinogen) and plasma (TEG with or without addition of tPA to induce fibrinolysis) in nine healthy volunteers before, during and after induction of experimental endotoxemia by means of a 4 h LPS-infusion (0.5 ng/kg/hour).

		Endotoxemia (n = 9)				
	Units	0 h	4 h	6 h	RM p-value	Tukey
**Multiplate**						
TRAPtest	U	125±24	85±19	89±21	**<0.001**	^a,b^
	U/platelet	0.59±0.15	0.45±0.08	0.51±0.11	**0.008**	^a^
ADPtest	U	79±15	57±12	55±10	**<0.001**	^a,b^
	U/platelet	0.37±0.10	0.30±0.04	0.32±0.06	0.064	
ASPItest	U	93±16	73±17	82±13	**0.028**	^a^
	U/platelet	0.42±0.10	0.37±0.07	0.45±0.04	0.117	
COLtest	U	84±14	64±14	70±13	**<0.001**	^a,b^
	U/platelet	0.40±0.10	0.34±0.04	0.40±0.06	0.060	
**Whole blood TEG**						
R	min	8.6±2.4	5.5±1.2	6.7±2.0	**0.003**	^a^
Angle	degrees	57±6	62±7	58±10	NS	
MA	mm	54±6	59±5	58±3	0.084	
G	dyn/cm2	6,010±1,232	7,229±1,487	6,928±934	**0.050**	^a^
LY30	%	0.7±0.8	0.7±1.1	0.8±1.1	NS	
LY60	%	3.5±2.6	3.7±2.9	4.3±3.7	NS	
CLT	min	136±38	71±16	151±5	**<0.001**	^a,c^
**Whole blood Functional Fibrinogen**						
R	min	5.6±1.2	5.2±1.3	5.7±1.5	NS	
Angle	degrees	29±8	32±9	32±11	NS	
MA	mm	14±2	12±2	12±3	**0.049**	^a^
G	dyn/cm^2^	787±151	688±157	671±210	0.051	
LY30	%	0±0	0±0	0±0	NS	
LY60	%	0±0	0±0	0±0	NS	
CLT	min	120±62	80±18	237±221	0.067	
**Plasma TEG**						
R	min	6.3±1.8	5.6±0.9	5.3±0.3	0.137	
Angle	degrees	45±13	52±8	56±7	**0.013**	^a,b^
MA	mm	19±3	19±3	20±3	0.151	
G	dyn/cm^2^	1,162±221	1,172±217	1,250±245	0.149	
LY30	%	0±0	0±0	0±0	NS	
LY60	%	0±0	0±0	0±0	NS	
CLT	min	124±36	117±40	114±40	NS	
**Plasma TEG +tPA**						
R _tPA_	min	6.2±2.1	5.6±0.8	5.1±0.5	0.178	
Angle _tPA_	degrees	44±14	53±8	57±6	0.011	^a,b^
MA _tPA_	mm	14±4	16±3	19±3	**<0.001**	^a,b,c^
G _tPA_	dyn/cm^2^	798±249	978±200	1,165±216	**<0.001**	^a,b,c^
LY30 _tPA_	%	51.1±26.2	25.2±29.0	0.1±0.4	**<0.001**	^a,b,c^
LY60 _tPA_	%	73.5±16.2	43.6±36.5	3.2±9.2	**<0.001**	^a,b,c^
CLT _tPA_	min	26±14	68±58	117±40	**<0.001**	^a,b,c^

Data are presented as means±SD. Data from volunteers were compared by repeated-measures analyses (RM) and Tukey post hoc tests: p<0.05 for 0 h vs. 4 h^a^, 0h vs. 6 h^b^ and 4 h vs. 6 h^c^. P-values <0.2 are shown and in bold if p<0.05.

Different platelet agonists were applied in the Multiplate tests: TRAPtest, thrombin-receptor activating peptide; ADPtest, ADP; COLtest, collagen; ASPItest, arachidonic acid; TEG, thrombelastography; R, reaction time; Angle, α angle; MA, maximum amplitude; G, shear elastic modulus strength; CLT, clot lysis time; Ly30/60, percent lysis 30/60 min after MA;

tPA, tissue-type plasminogen activator. NS, non-significant.

**Figure 1:**
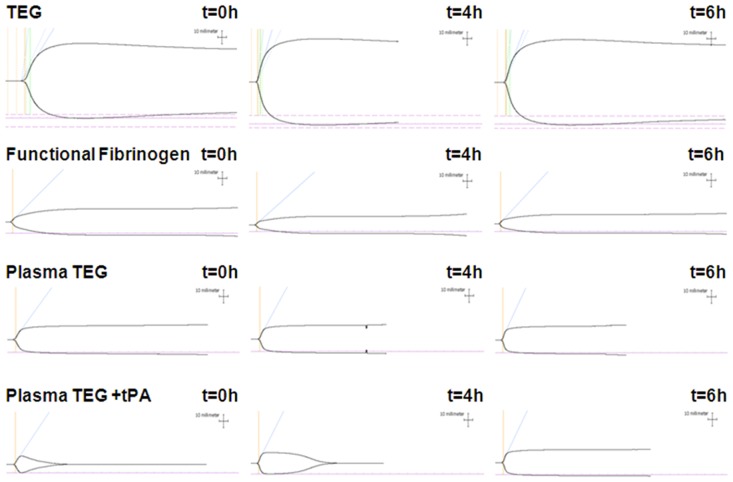
Representative profiles of TEG and modified TEG analyses in healthy volunteers before (t = 0), during (t = 4 h) and after (t = 6 h) experimental endotoxemia induced by a 4-hour continuous intravenous infusion of purified *Escherichia coli* LPS (infusion rate 0.5 ng/kg/hour). TEG and Functional Fibrinogen were analyzed in whole blood whereas plasma TEG was analyzed in plasma with or without addition of 1.8 nM tPA to induce lysis of the clot i.e., allowing extended evaluation of clot resistance to fibrinolysis.

Secondary hemostasis (fibrin-platelet clot formation in whole blood) also changed during and after endotoxemia as evaluated by standard and modified TEG® analysis (representative profiles displayed in Figure 1): TEG R-time decreased, G increased and CLT decreased during endotoxemia, indicating combined increases in coagulation factor activity, clot strength and clot lysis, respectively. In contrast, Functional Fibrinogen (whole blood, blocked platelet GPIIb/IIIa receptors) MA decreased after endotoxemia indicating reduced fibrinogen level and fibrin clot strength.

The calculated platelet contribution to TEG MA (platelet MA) increased during endotoxemia (p = 0.006) from 41±5 mm at baseline (0 h) to 47±5 mm at 4 h and 46±2 mm at 6 h (both p<0.05 compared to 0 h), probably explaining the discrepant increase in TEG MA and decline in FF MA (Table 2). Since platelet count decreased during endotoxemia, the platelet MA per platelet also increased (p<0.001) from 0.19±0.04 mm/10^9^ platelets at baseline (0 h) to 0.25±0.04 mm/10^9^ platelets at 4 h and 0.27±0.05 mm/10^9^ platelets at 6 h (both p<0.001 compared to 0 h).

There was no evidence of endogenous heparinization as evaluated by the difference between TEG and heparinase-TEG (TEG minus heparinase-TEG) [38]: delta R at 4 h −0.1±0.2 and 6 h 0.4±1.2; delta angle at 4 h −0.5±3.9 and 6 h 0.3±8.3; delta MA at 4 h −3.0±5.4 and 6 h −4.4±4.0 (all non-significantly different from zero indicating that TEG and heparinase-TEG were comparable).

### Hemostatic response in plasma

In contrast to the findings in whole blood, TEG Angle increased and TEG+tPA MA and G increased in plasma during and after endotoxemia indicating increased clot velocity and strength/firmness, respectively (Table 2). Furthermore, plasma TEG+tPA Ly30/60 decreased considerably whereas CLT increased during and after endotoxemia, indicating increased resistance towards the tPA-induced in vitro clot lysis (Figure 1 and Table 2).

### Correlations between plasma fibrinogen, platelet count and TEG MA

The main determinants of TEG/ROTEM clot strength are fibrinogen and platelets (in addition to FXIII which we did not measure) [68]–[71]; we therefore investigated correlations between these variables and TEG MA during endotoxemia. At baseline, fibrinogen correlated significantly positively with TEG MA (r = 0.83, p = 0.006) whereas platelets did not (r = 0.14, p = NS). However, during (4 h) and after (6 h) endotoxemia, the contribution of fibrinogen to TEG MA decreased and became non-significant (r = 0.32 and r = 0.25, both p = NS) whereas the contribution of platelets to TEG MA increased non-significantly at 4 h (r = 0.55, p = 0.128), indicating increased platelet reactivity in the context of declining platelet count. After endotoxemia (6 h), TEG MA and platelet count did not correlate significantly (r = 0.18, p = NS).

## Discussion

The main finding of the present study was that experimental endotoxemia, along with induction of SIRS, resulted in a hemostatic response in whole blood with reduced primary but enhanced secondary hemostasis, enhanced early fibrinolysis and fibrinogen consumption followed by inhibition of fibrinolysis. In plasma, however, endotoxemia resulted in a progressive increase in clot resistance towards tPA-induced fibrinolysis indicating that the early LPS/inflammation-induced increase in fibrinolytic activity may be mediated by cellular elements in the whole blood.

Sepsis is associated with vascular dysfunction and coagulopathy that may progress from an initially normal coagulation profile to hypercoagulability, hyperfibrinolysis and ultimately hypocoagulability with increasing disease severity [11]–[13], [18]. This has been documented by TEG/ROTEM and has been reproduced in studies using experimental human [23] and animal [25]–[27] models of endotoxemia [23], [25]–[27]. Also, platelet aggregation is profoundly reduced in septic patients [19]–[22] and decreases with disease severity [20]–[22], which has also been reproduced in experimental human [23], [24] and animal [28], [29] models of endotoxemia.

In the present study, experimental endotoxemia, by means of a 4-hour infusion of 0.5 ng/kg/hour LPS, reduced primary hemostasis (platelet aggregation, reduced by Multiplate) and enhanced secondary hemostasis (clot formation) in whole blood (reduced R time (enhanced coagulation initiation) and increased G (enhanced clot strength) at 4 h by TEG) in accordance with previous findings [23]–[25], [27]–[29]. During endotoxemia, fibrinolysis was enhanced (increased breakdown of the platelet-fibrin clot, CLT reduced at 4 h by TEG), which probably explains the observed reduction in the functional fibrinogen level (reduced strength of the fibrin clot, MA reduced at 4 h by FF). However, after endotoxemia, fibrinolysis was inhibited (increased CLT at 6 h by TEG), in accordance with previous findings in human [23] and animal [25] studies of experimental endotoxemia.

In contrast to the reduction in platelet aggregation, platelet clot formation increased (the pure contribution of platelets to TEG clot strength, platelet MA increased at 4 h and 6 h) both in total and on a per platelet basis (i.e. each platelet became more reactive), in accordance with the notion that the platelet fibrinogen receptor activity (GPIIb/IIIa) is enhanced in early sepsis [50].

Given that thrombosis may represent a critical component of innate immunity [72], the hemostatic changes observed in response to endotoxemia probably reflect part of a coordinated immune response. Thus, there is emerging evidence that platelet function goes far beyond hemostasis and that platelets are critically involved in and modulators of host defense and immune function [73]–[76]. Hence, Yaguchi and colleagues [21] suggested almost 10 years ago that sepsis induces a redistribution of platelet function from hemostasis toward other functions like e.g. vascular healing. We recently reported of an association between excessive sympathoadrenal activation and reduced hemostatic function of platelets in trauma patients [77], [78] suggesting that catecholamines may promote a switch in platelet function from hemostasis to e.g. immunomodulation [42], [76]. We infer that the change in hemostatic platelet function in critical illness may serve to sustain platelet circulation (avoiding consumption), thereby allowing platelets to exert other functions like e.g., vascular healing [21] and immunomodulation [76].

The oppositely directed responses in primary and secondary hemostasis observed in the present study probably reflect that we investigated an early hemostatic response to acute inflammation, in which it is well documented that secondary hemostasis is enhanced rather than reduced [11]–[13], [23], [25], [27].

In contrast to the early increase (4 h) in and later downregulation (6 h) of fibrinolysis observed in whole blood by TEG, the resistance of the fibrin clot towards tPA-induced fibrinolysis increased progressively both during (4 h) and after (6 h) endotoxemia. This discrepancy indicates that blood cells and/or blood cell-derived microparticles and/or molecules enhance fibrinolysis in acute inflammation in accordance with the notion that inflammatory and endothelial cells and their derived microparticles can promote fibrinolysis [53]–[55]. Though platelets in some studies have been reported to enhance clot resistance towards fibrinolysis [51] it cannot be excluded that platelets under conditions characterized by systemic inflammation accompanied by endothelial activation and damage may change their phenotype towards a more pro-fibrinolytic one to avoid clot formation in the microvasculature thereby ensuring adequate organ perfusion. Given that degranulation of platelets is a highly regulated process [79], [80], it is also tempting to speculate that thrombocytopenia, depending on the context, may both enhance and reduce fibrinolysis. Besides platelets, other blood cells, leukocytes in particular, may also through mechanisms such as release of neutrophil elasase, enhance fibrinolysis and thereby contribute to a discrepant response in plasma and whole blood [57]. Finally, this discrepancy emphasizes the importance of evaluating hemostasis in whole blood, opposite plasma, while also taking the concurrent state of the vascular endothelium (pro- vs. anticoagulant) into account since the states of the fluid (circulating blood) and solid (endothelium) phases of the vascular system may, from a systems biology perspective, counterbalance each other [42]. Thus, in conditions with systemic inflammation and/or coagulation activation, progressive hypocoagulability and fibrinolysis of the circulating blood may serve to keep a progressively more damaged and procoagulant microvasculature open in order to maintain perfusion of critical organs [42]. This notion is in accordance with the progressive hypocoagulability and fibrinolysis reported in septic patients [11]–[13], [18] and it also agrees with the finding of hyperfibrinolysis in trauma [77], [81]–[90], cardiac arrest [91] and major surgical [92] patients and with the finding of enhanced protein C activation in patients with septic shock [93], during the reperfusion phase after cardio pulmonary bypass [94] and in cardiac arrest [95] or severely injured [77] patients. Despite thrombosis being a critical player in innate immunity [72], it is notable that several of the endothelial derived molecules that promote hypocoagulability exert potent antiinflammatory and cytoprotective functions [96]–[99] that may ultimately generate at survival advantage in critically ill patients.

We found no evidence to suggest that endotoxemia induces endogenous heparinization. This contrasts previous findings in septic [32]–[37] and trauma [38] patients. This discrepancy is likely explained by the presence of endothelial damage and hence glycocalyx degradation in patients with severe sepsis [5], [6], [100], which cannot be reproduced in human experimental endotoxemia since the LPS-doses required to mimic severe sepsis with regard to endothelial damage and/or organ dysfunction are unsafe and ethically unacceptable [1]–3.

The present study has several limitations. Firstly, only young male volunteers were investigated in the experimental part and consequently a possible age and/or gender difference in response to endotoxemia was not evaluated. Furthermore, the low number of volunteers investigated increases the risk of introducing both type I and II errors, emphasizing that the findings herein should be confirmed in a larger studies. Finally, though the administered noradrenaline infusion [65] was assumed completely metabolized before blood sampling [66], it cannot be excluded that the infused noradrenaline influenced platelet number and function as catecholamines including noradrenaline influence both platelet adhesion and activation [45].

In conclusion, induction of experimental endotoxemia by means of a 4-hour LPS-infusion at 0.5 ng/kg/hour induced a hemostatic response comparable to that observed in early sepsis, with reduced primary but enhanced secondary hemostasis, enhanced early fibrinolysis and fibrinogen consumption followed by downregulation of fibrinolysis. In plasma, however, endotoxemia resulted in a progressive increase in clot resistance towards tPA-induced fibrinolysis. We infer that the discrepant fibrinolytic response observed in plasma and whole blood reflects a critical role of platelets and/or other blood cells in the vasculo-fibrinolytic response to acute inflammation in accordance with the finding here of significantly altered platelet function in response to endotoxemia.
